# Transcriptomics reveals pallial and subpallial subdivisions of the mouse medial amygdala

**DOI:** 10.1007/s00429-026-03076-x

**Published:** 2026-02-02

**Authors:** Gloria Fernández, Luis Puelles, Eduardo Pons-Fuster, Ramón Pla, Elena Garcia-Calero

**Affiliations:** Departamento de Anatomía Humana y Psicobiología, Facultad de Medicina, Universidad de Murcia e Instituto Murciano de Investigación Biosanitaria IMIB-Pascual Parrilla, Murcia, Spain

**Keywords:** SnRNAseq, Limbic system, Amygdalar radial model, Pallial amygdala

## Abstract

The medial amygdala plays a key role in social brain networks supporting affiliation behavior. This whole domain is widely considered a subpallial (GABAergic) amygdalar subdivision, although it is composed of a mixture of cells of different origins, including populations migrated from the hypothalamus, the prethalamic eminence and the neighboring pallial amygdala. Specifically, the ventral part of the medial amygdala has a high percentage of glutamatergic cells. We developed a transcriptomic study (snRNAseq) to examine the pallial versus subpallial ascription of the mouse medial amygdala cells. We detected high molecular similarity between the ventral region of the medial amygdala and the pallial *anterior* amygdalar radial unit (classical anterior basomedial nucleus, BMA, and anterior cortical nucleus, ACo), in contrast with the GABAergic profile of the posterodorsal region of the medial amygdala, and the central and intercalated amygdalar nuclei. To verify the possible pallial origin of the ventral medial amygdala, we also analyzed pallial and subpallial marker genes in this region during development and in adult specimens. In addition, we labeled radial glia at perinatal stages to reconstruct the radial domain to which the ventral medial amygdala belongs. The results are consistent with a pallial nature of the ventral medial amygdala, while the posterodorsal medial amygdala is subpallial.

**Supplementary Information:**

The online version contains supplementary material available at 10.1007/s00429-026-03076-x.

## Introduction

The mammalian medial amygdala (MeA) is widely held to be a component of the subpallial amygdala, jointly with the anterior amygdala (AA), central amygdala (CeA) and intercalated nuclei of the amygdala (IcA) (Krettek and Price 1978; De Olmos et al. 1985; Olmos et al. 2004; Alheid et al. 1995; Swanson and Petrovich 1998; Martínez-Garcia et al. 2012; Olucha-Bordonau et al. 2015; Medina et al. 2017). The MeA region is described functionally as a part of the vomeronasal system, implicated in sexual, parenting, and agonistic behaviors. As a node in this system, it integrates vomeronasal and other stimuli (for example, olfactory ones) with various hypothalamic responses (Choi et al. 2005; Pro-Sistiaga et al. 2007; Martinez-Marcos 2009; Abellan et al. 2013; Chen et al. 2019; Raam and Hong 2021; Guo et al. 2023; Prakash et al. 2024). Rodent atlases and many publications usually identify anterior/posterior and dorsal/ventral partitions of the MeA domain (MeAD, MeAV, MePD, MePV, De Olmos et al. 2004; Martinez-Garcia et al. 2012; Olucha-Bordonau et al. 2015; Paxinos and Franklin 2019). There is apparently some segregation of functions between these parts (Choi et al. 2005).

Neurotransmitter markers reveal numerous GABAergic cells in the medial amygdala, leading to the original ascription of this region to the subpallium (Swanson and Petrovich 1998). However, studies focused on the posteroventral subdivision (MePV) concluded that about 70% of its cells are non-GABAergic (Choi et al. 2005; Bian et al. 2008; Keshavarzi et al. 2014). This result reopened the issue of the intrinsic pallial versus subpallial nature of the different parts of the MeA, which combines with the potential existence of tangentially migrated GABAergic or glutamatergic neurons in variable proportions. There is developmental evidence that glutamatergic and GABAergic cell populations originated from different pallial, subpallial or hypothalamic sources mix within the mammalian MeA (Hirata et al. 2009; Garcia-Moreno et al. 2010; Carney et al. 2010; Puelles et al. 2016; Lischinsky et al. 2017; Ruiz-Reig et al. 2018; Garcia-Calero et al. 2021). Hypothalamic *Otp*-expressing and preoptic *Shh*-expressing cells invade the mouse MeA at early developmental stages (Garcia-Moreno et al. 2010; Carney et al. 2010). In addition, the MePV subdomain receives *Pax6*-positive cells whose source remains unclear (Ruiz-Reig et al. 2018; suggested their tangential migration from the prethalamic eminence -PThE, where only the ventricular zone expresses this gene- but they also may originate by radial migration from the caudal part of the lateral ganglionic eminence -CGE; both PThE and CGE express *Pax6* in the ventricular zone and the CGE/LGE produces migrating *Pax6*-positive neurons). GABAergic neurons from the subpallial diagonal histogenetic area that co-express somatostatin cells (Puelles et al. 2016), and glutamatergic *Ebf3*-positive cells from the pallial telencephalic caudal pole (Ruiz-Reig et al. 2018) also invade the MeA territory. Garcia-Calero and Puelles (2021) identified a tangential migration of glutamatergic *Lhx9*-positive neurons from the anteromedial amygdalar nucleus into the ventral MeA (both MeAV and MePV). These results challenge our understanding of MeA development and raise the issue of its pallial versus subpallial primary nature.

We proposed a new morphological model for the rodent pallial amygdala based on its natural radial structure (i.e., correcting the widespread literature tradition to describe amygdalar structure on oblique sectioning planes; Garcia-Calero et al. 2020). In this model, the pallial amygdala is divided into four main complexes of radially stratified nuclei: the *anterior*, *lateral*, *basal* and *posterior* radial domains or units. Each radial unit is stratified in periventricular, intermediate and superficial strata. In our recent snRNAseq transcriptomic study of the pallial amygdala (Fernández et al. 2025) we observed collaterally molecular similarity between the glutamatergic neurons of the *anterior* radial pallial domain and cells within the ventral division of MeA; this result contrasts with a different distinctly GABAergic profile of cell populations in the dorsal MeA and other subpallial regions (unpublished observations). In the present work, we report a transcriptomic analysis by snRNAseq experiments focused on the MeA and neighboring areas, aiming to clarify the distribution of pallial versus subpallial characteristics. We also mapped developmental expression of *Dlx5* and *Slc17a6* (GABAergic versus glutamatergic markers, respectively), together with other local markers of interest, such as *Pax6*. To clarify the radial dimension of the mouse MeA, we labelled radial glia packets at perinatal stages using pial DiI injections. According to these combined studies, the MeV is a distinct radial pallial subdivision of the MeA (note we include in MeV the conventional MeAD, MeAV and MePV parts), whereas the remaining posterodorsal subdivision (MePD) is clearly subpallial.

## Results

### Transcriptomics of medial amygdala in the context of the whole amygdala and its surroundings

We started by examining a single-nuclei map of the entire amygdala region (pallial and subpallial) including portions of neighboring GABAergic subpallial areas. This data corresponds to a previous snRNAseq dataset recently produced by our group to study the pallial amygdala (Fernández et al. 2025; the Seurat object merged four adult samples of the amygdala region, two males, AM1 and AM2, and two females, AF1 and AF2, see Materials and Methods in Fernandez et al., 2025, and present Results and Material and methods). We selected data on amygdalar and GABAergic neurons by subsetting the nuclei in these areas (identified as APall/GABA in Fernández et al. 2025; their Fig. 2e) from the adult Seurat object. We obtained an object with 6.979 high quality nuclei with all samples represented in all clusters (S1a and b).

**Fig. 1:**
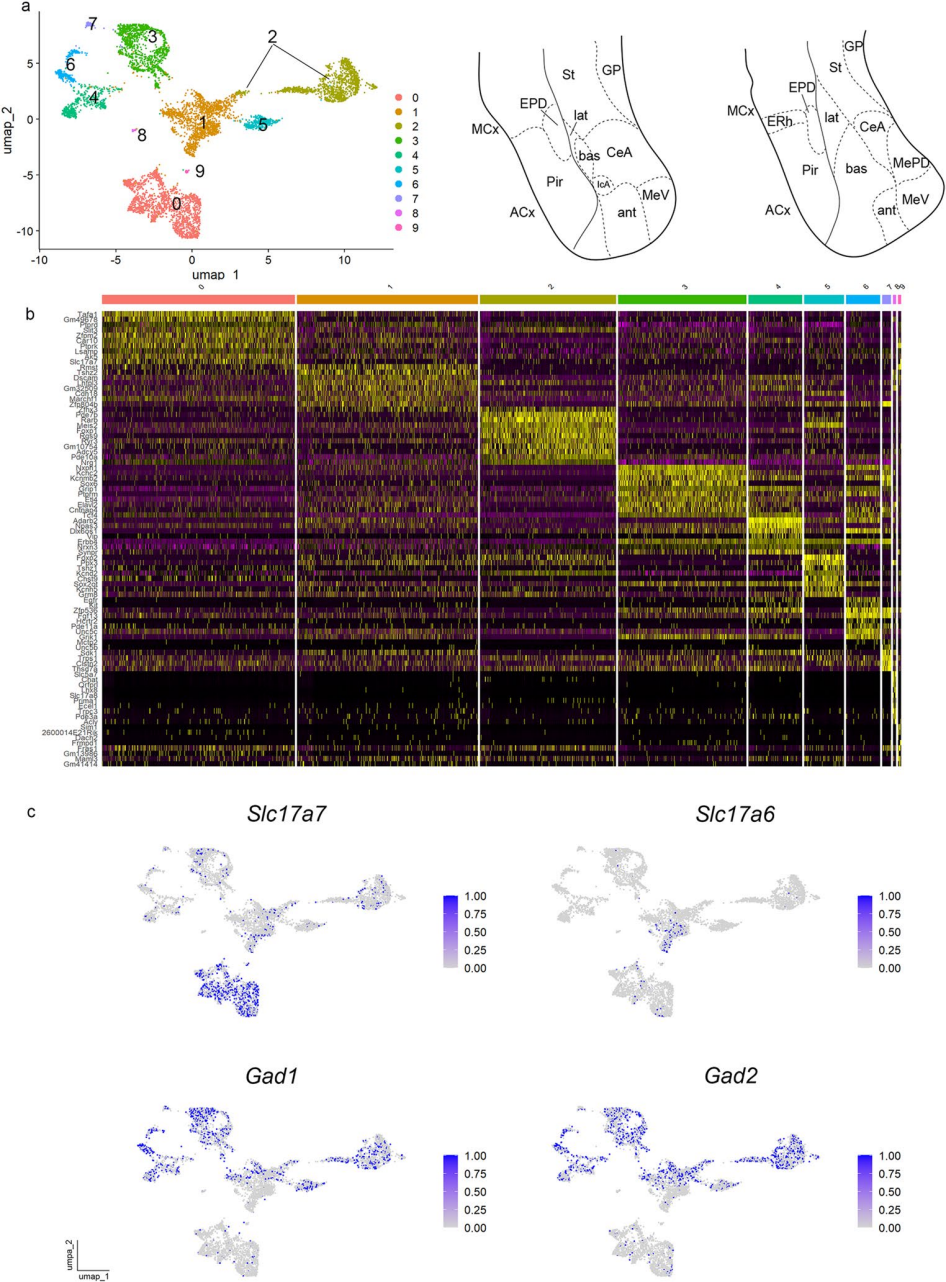
Transcriptomics of medial amygdala and neighboring regions. (a) Uniform Manifold Approximation and Projection for Dimension Reduction (UMAP) plot of the adult pallial amygdala and GABAergic-Seurat object (6.979 nuclei) colored by clusters. Scheme of the temporal pole of the telencephalon (b) Heatmap of the 8 top genes differentially expressed among the 10 clusters at resolution 0.2. (c) UMAP plots for *Slc17a7*, *Slc17a6*, *Gad1* and *Gad2* in the pallial amygdala and GABAergic-Seurat object

**Fig. 2:**
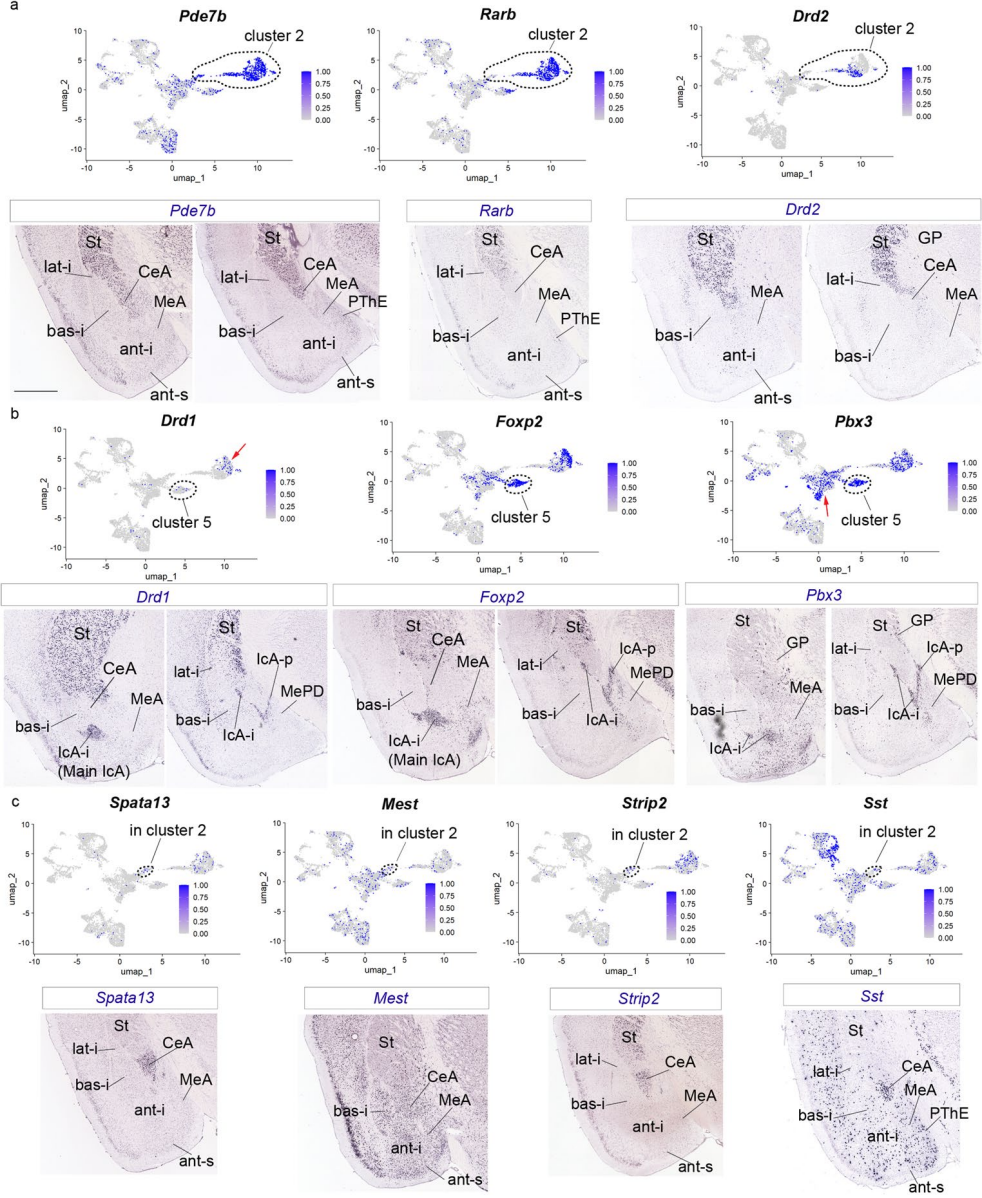
Gene markers for striatum, central amygdala and intercalated amygdala nuclei. (a) UMAP plots for *Pde7b*, *Rarb* and *Drd2* of the pallial amygdala and GABAergic-Seurat object, and *in situ hybridization* for these genes. Cluster 2 was highlighted with an outline in the UMAP plots. (b) UMAP plots for *Drd1*, *FoxP2* and *Pbx3* of the pallial amygdala and GABAergic-Seurat object, and in situ hybridization for these genes. Cluster 5 was highlighted with an outline in the UMAP plots; red arrow point to cluster 2 in the *Drd1* UMAP plot, and to cluster 1 in *Pbx3* UMAP plot (c) UMAP plots for *Spata13*, *Mest*, *Strip2* and *Sst* of the pallial amygdala and GABAergic-Seurat object, and in situ hybridization for these genes. A region of cluster 2 representing CeA was highlighted with an outline in the UMAP plots. The in situ hybridization figures of Fig. 2 were downloaded from AMBA. Coronal plane. Scale bar 930 μm

**Fig. 3:**
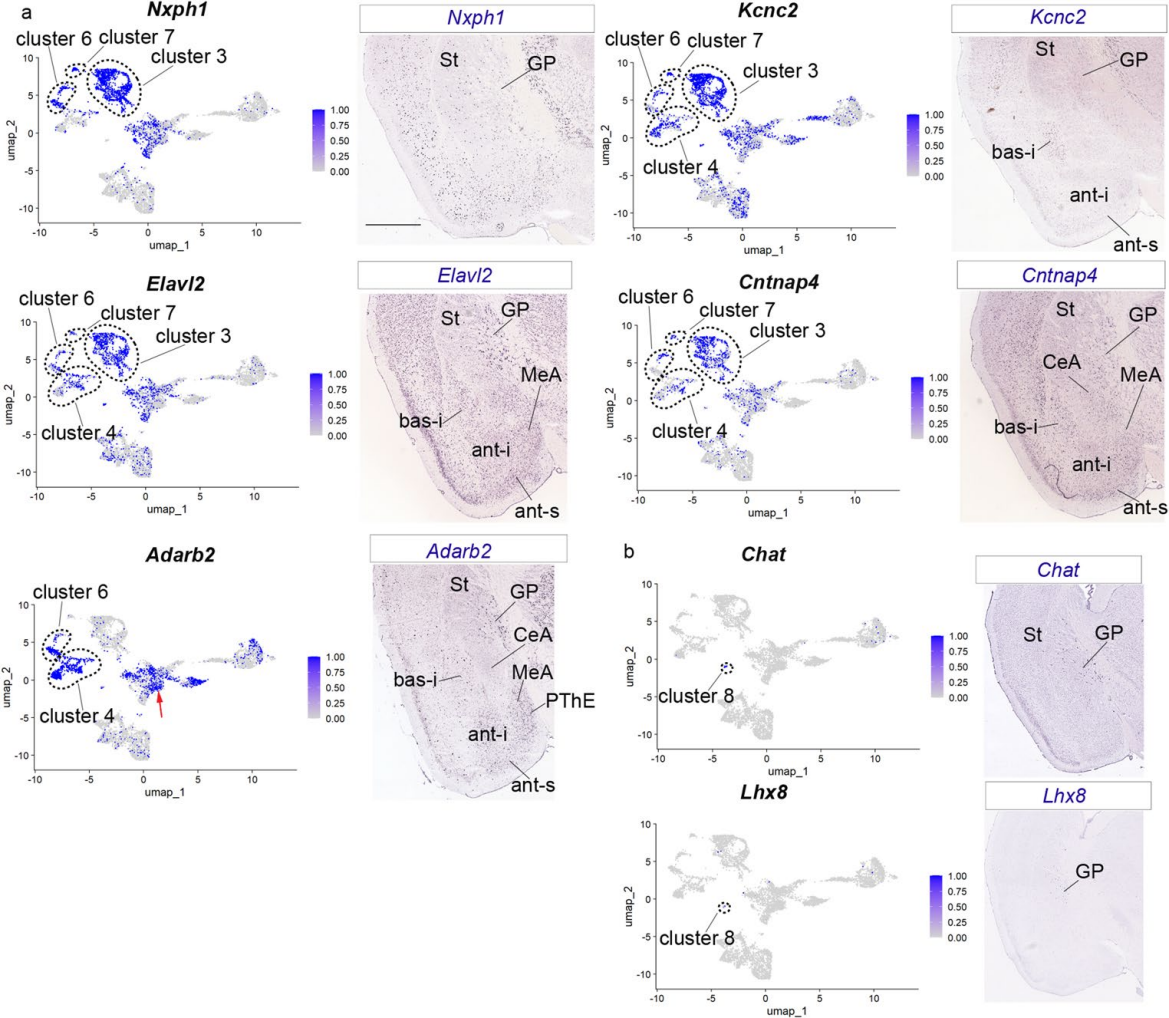
Gene markers for pallidal region. (a) UMAP plots for *Nxph1*, *Kcnc2*,* Elavl2*,* Cntnap4*, and *Adarb2* of the pallial amygdala and GABAergic-Seurat object, and in situ hybridization for these genes. Clusters 3, 4, 6, 7 were highlighted with an outline in the UMAP plots; red arrow in the Adarb2 UMAP plot point to cluster 1. (b) UMAP plots for *Chat*, and *Lhx8* of the pallial amygdala and GABAergic-Seurat object, and in situ hybridization for these genes. Cluster 8 was highlighted with an outline in the UMAP plots. The in situ hybridization figures of Fig. 3 were downloaded from AMBA. Coronal plane. Scale bar 930 μm

Cluster resolution was optimized aiming for UMAP clusters that separated groups of molecularly different cells (to be identified numerically); the chosen cluster resolution was 0.2 (Fig. 1a; compare cluster resolutions of 0,05, 0,1 and 0,15 in S1c). At cluster resolution 0.2, we detected 10 clusters in our pallial amygdala/GABAergic object (illustrated via Uniform Manifold Approximation or UMAP plot, together with the scheme of the telencephalic temporal pole, Fig. 1a). To detect differential gene expression among clusters we used the “FindAllMarkers” function from Seurat-5.1.0 (heatmap of differentially expressed genes, DEGs, in Fig. 1b; Hao et al. 2024). First, we checked the expression of glutamatergic (*Slc17a6* and *Slc17a7*) and GABAergic (*Gad1* and *Gad2*) markers in our pallial amygdala/GABAergic dataset (see UMAP plots for these genes in Fig. 1c), to distinguish between pallial and subpallial clusters. To annotate the 10 clusters, we also studied the expression of the identified DEGs by in situ hybridization results from the Allen Adult Mouse Brain Atlas (AMBA) and also used its AGEA tool to find additional useful markers for these areas (Figs. 2 and 3 and S2). For the nomenclature of pallial amygdalar radial subdivisions we followed that adopted in Fernandez et al. (2025) and employed towards topographic description an abbreviated identity tag of the form “lat-i”; this corresponds to the abbreviation of the radial domain name (chosen among *ant*; anterior; *post*; posterior; *lat*; lateral; *bas*; basal) separated by a hyphen from the abbreviation of the relevant radial stratum (-*p*; periventricular; *-i*; intermediate; -*s*; superficial). The ’lat-i’ tag thus means ‘the intermediate stratum of the lateral radial unit’.

Cluster 0 was enriched in *Slc17a7 and Zfpm2* (Fig. 1b, c), two markers characteristic of the pallial amygdala, expressed in its *lateral*, *basal* and *posterior* radial domains (Fernández et al. 2025). Cluster 1 was enriched instead in *Slc17a6*, a typical glutamatergic marker for the *anterior* radial domain of the pallial amygdala (Fernandez et al. 2025) though it also displayed GABAergic transcripts (Fig. 1c).

Clusters 2–9 were rich in the GABAergic markers *Gad1* and *Gad2* and corresponded to diverse amygdalar and non-amygdalar subpallial regions (Fig. 1c).

#### Striatal clusters

Cluster 2 expressed the markers *Pde7b*, *Rarb*, *Drd2*, *Drd1*, *Foxp1*, *Meis2*, *Rgs9* and *Adcy5* which are striatal markers (St; cluster 2 was highlighted with an outline in the UMAP plots, Fig. 2a and S2a; in Fig. 2b, cluster 2 is pointed to with a red arrow in the *Drd1* UMAP cluster). As expected, these genes were expressed mainly in striatal domain; in particular, *Rarb*, *Rgs9 and Adcy5* were very specific to the striatal formation. *Drd1* and *Drd2* showed a distinct segregation of their expression in these UMAP plots (Fig. 2a, b). Among this group of markers, *Pde7b*, *Meis2* and *Adcy5* clearly labelled specifically the central amygdalar nuclear complex (medial, lateral and capsular parts), which is classically considered to belong to the amygdalar caudal end of the striatum (St; CeA; in situ hybridization from AMBA in Fig. 2a and S2a).

Jumping to data on other striatal regions, cluster 5 showed high expression of *Drd1*, *FoxP2*, *Pbx3*, *Tshz1* and *Erbb4* corresponding to the complex of the intercalated amygdalar islets (IcA), a poorly understood caudal striatal region that lies at the boundary between the striatum and the lateral and basal pallial amygdala portions (cluster 5 was highlighted with an outline in the UMAP plots, Fig. 2b and S2b). The IcA formation forms typically a tridimensional curved network of cell islands separated by cell-poor spaces that limit dorsorostrally and ventrorostrally the intermediate nuclei of the *lateral* and *basal* radial domains (lat-i, basal-i, corresponding to the classic lateral, L, and anterior basolateral, BLA nuclei). We identified during the present study its caudally lying periventricular subregion, laterally adjacent to the MePD (IcA-p; MePD; Fig. 2b and S2b; rodent brain atlases usually identify this locus as STIA or amygdalar bed nucleus of the stria terminalis; Paxinos and Franklin 2019; see Discussion). Apart of the IcA-p, the intermediate network of IcA cell islands constitutes the classical intercalated nuclei of the amygdala (IcA-i; in situ hybridization from AMBA in Fig. 2b and S2b; note the largest of such intercalate islands – the principal or main IcA typically appears ventromedially to the rostralmost part of the basal-i nucleus in the mouse).

Interestingly, cluster 5 related to the IcA complex lies rather close to cluster 1 (related to the *anterior* pallial amygdalar unit), being at some distance from the striatal cluster 2 (Fig. 1a). Nearby there appears likewise a group of cells of cluster 2 that relates specifically to the central subpallial amygdala; surprisingly, these cells are also adjacent to cluster 1 (Fig. 1a; black lines in Fig. 2c). We checked these relationships with the markers *Spata13*,* Strip2*,* Mest* and *Sst* (AGEA tool results) that localize to the central amygdala in the amygdalar striatal sector. In our figures, *Spata13*,* Strip2*,* Mest* and *Sst* clearly label selectively the CeA (in situ hybridization data from AMBA; Fig. 2c). We observed additional transcripts in the main striatal domain in our UMAP plots for these genes (the region of cluster 2 that reprsents the central amygdalar cells was highlighted with an outline). Taking into consideration previous observations on the striatal domain (including expression of the *Pde7b*, *Meis2* and *Adcy5* markers), we conclude that CeA is a group of cells belonging characteristically to cluster 2 but lying molecularly close to cluster 1.

#### Pallidal clusters

Clusters 3, 4, 6 and 7 are also subpallial but display pallidal markers (based on the globus pallidus; GP) such as *Nxph1*, *Kcnn2*, *Elavl2*, *Cntnap4*, *Adarb2*, *Clstn2*, *Ths7a* (clusters were highlighted with an outline, Fig. 3a and S2c). Cluster 8 corresponds to the small population of *Chat/Lhx8* positive cells found interstitially within the striatum (supposedly representing migrated pallidal interneurons) or present within pallidal and diagonal parts of the subpallium (cluster 8 was highlighted with an outline in UMAP plots in Fig. 3b; see Puelles et al. 2023). The expression pattern of these genes shows positive cells localized mainly in the pallidal area (Fig. 3b).

#### Hypothalamic cells

Cluster 9 represents a glutamatergic *Sim1-*expressing population that has been described migrate tangentially from the alar paraventricular hypothalamus into the subpallial amygdala, after incorporating into the amygdalar migration stream of the NLOT2 nucleus (cluster 9 in Fig. 1a; heatmap in Fig. 1b; see Garcia-Calero et al. 2021).

#### MeA cluster

**Fig. 4:**
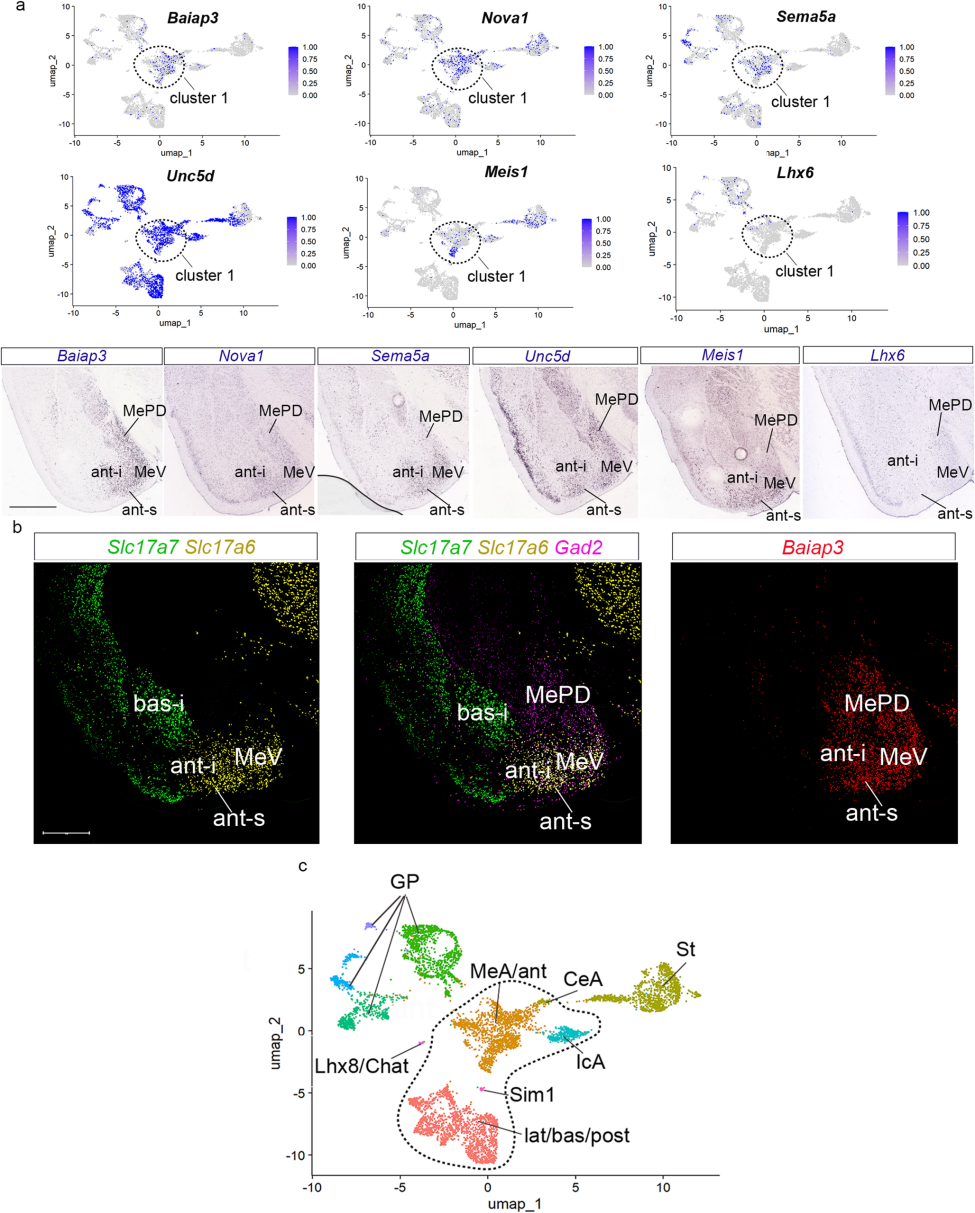
Gene markers for medial amygdala and *anterior* radial unit. (a) UMAP plots for *Baiap3*, *Nova1*,* Sema5a*,* Uncd5*,* Meis1* and *Lhx6* of the pallial amygdala and GABAergic-Seurat object, and in situ hybridization for these genes. Cluster 1 was highlighted with an outline in the UMAP plots. (b) Multiplexed fluorescent in situ hybridization assays for *Slc17a7*,* Slc17a6*,* Baiap3 and Gad2* in the amygdalar region. Coronal plane. Scale bars 500 μm. (c) UMAP plot of the Seurat object annotated with the different subpallial and pallial regions in the temporal telencephalic pole. Amygdalar clusters were outlined to highlight it. The in situ hybridization figures of Fig. 4 were downloaded from AMBA. Coronal plane. Scale bar 930 μm

Markers correlating more or less specifically with the MeA are found in cluster 1 (Fig. 4a). We previously reported on some molecular similarities observed between the *anterior* radial domain of the pallial amygdala and the MeA (Garcia-Calero et al. 2020; Fernandez et al. 2025). We re-checked the expression of the following *anterior* radial domain markers in our present dataset: *Baiap3*, *Nova1*, *Sema5*, *Unc5d*, *Adarb2*, *Meis1*, *Pbx3.* Some of these markers are separately described as present in other clusters but also in cluster 1; this aspect was pointed out with red arrows in the UMAPs (e.g., red arrow in the *Pbx3* UMAP plot in Fig. 2b; red arrow in the *Adarb2* UMAP plot in Fig. 3a; the other more selective marker genes were identified highlighted with an outline in the UMAP plots in Fig. 4a). We also analyzed the expression of *Lhx6* as a marker of MeA (Fig. 4a).

We focused on the expression of *Baiap3* compared with the glutamatergic markers *Slc17a6* and *Slc17a7*, or the GABAergic marker *Gad2* (Fig. 4b). We observed that *Slc17a6* is expressed in the *anterior* radial complex and the MeV, whereas *Gad2* expression predominates in the MePD (ant; MeV; MePD; Fig. 4b). *Slc17a7*, on the other hand, displayed low expression in the *anterior* radial complex or medial amygdala (ant; MeV; MePD; Fig. 4b). In contrast, *Baiap3* expression extends throughout the whole medial amygdala and appears also in the *anterior* amygdalar pallial complex (ant; MePD, MeV; Fig. 4b).

#### Transcriptomic closeness of MeA and the *anterior* pallial radial unit

We concluded that cluster 1 examined at resolution level 0.2 corresponds jointly to the *anterior* radial domain and to the glutamatergic and GABAergic cell populations of the MeA (see UMAP plots with cluster annotations for amygdalar and GABAergic areas in Fig. 4c).

The *anterior* pallial radial domain of the amygdala, which clusters together with the MeA, displays otherwise considerable molecular similarity with other regions of the amygdalar pallial complex, such as the *lateral*, *basal* and *posterior* radial complexes (Fernández et al. 2025). When studied at resolution 0.2, the CeA and IcA amygdalar nuclei, held to be subpallial, are molecularly differentiable from the MeA but also lie close to the cluster 1 formed by the *anterior* pallial radial domain and MeA. The next section examines closer the cell populations in cluster 1 at higher resolution.

### SnRNAseq experiments on the subsetted cluster 1

**Fig. 5:**
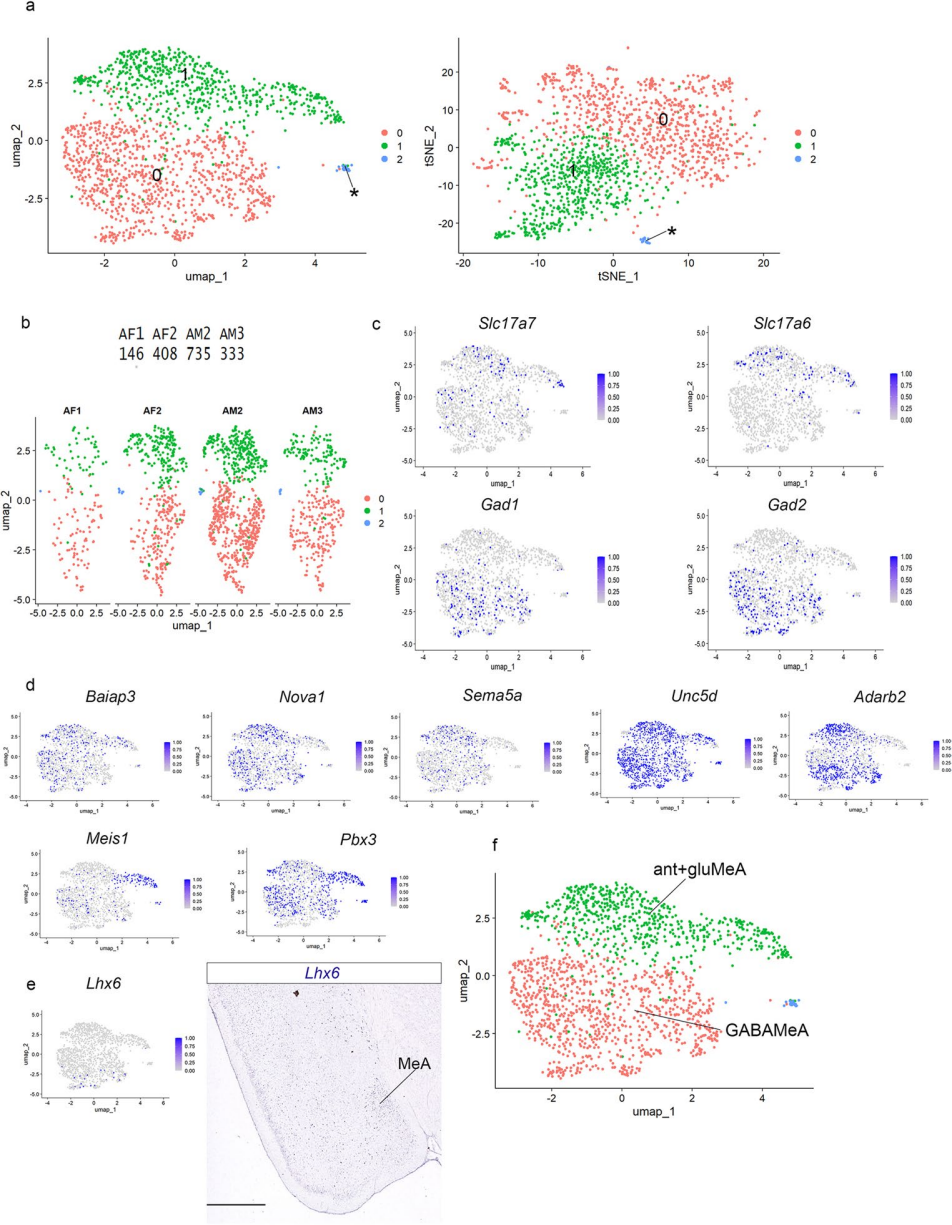
Transcriptomics of medial amygdala and the *anterior* radial unit. (a) UMAP and t-SNE plots of the adult *anterior* radial unit and MeA-Seurat object (1.622 nuclei) colored by clusters. (b) UMAP plot of *anterior* radial unit and MeA-Seurat object split by sample. (c) UMAP plots for *Slc17a7*, *Slc17a6*, *Gad1* and *Gad2* of the *anterior* radial unit and MeA-Seurat object. (d) UMAP plots for *Baiap3*,* Nova1*,* Sema5a*,* Uncd5*,* Adarb2*,* Meis1*,* Pbx3*, markers of the amygdalar *anterior* radial complex. (e) UMAP plot for *Lhx6* and in situ hybridization downloaded from AMBA. Coronal plane. Scale bar 930 μm. (f) UMAP plot of the *anterior* radial unit and MeA-Seurat object annotated for the MeA posterodorsal and ventral subdivisions, and the *anterior* radial amygdalar domain. ant + gluMeA: anterior radial domain and glutamatergic amygdalar cluster; GABAMeA: GABAergic amygdalar cluster. MeA: medial amygdala

Next, we subsetted the medial amygdala/*anterior* radial cluster (cluster 1 in Fig. 1a; MeA/ant in Fig. 4c) and obtained an object of 1.622 high quality nuclei. We evaluated its cluster structure at three resolution levels: 0.05, 0.15 and 0.3 (S3). At resolution 0.15, we obtained two main clusters (clusters 0 and 1), with the four samples (AM1, AM2, AF1, AF2) represented in both clusters (UMAP plot and tSNE plot in Fig. 5a, UMAP split by sample in Fig. 5b; there was also a very small third cluster whose significance was not explored further; asterisk; Fig. 5a, S3). We checked the expressions of glutamatergic, GABAergic and various *anterior* radial pallial domain markers in this dataset (Fig. 5c and d). At resolution 0.15, cluster 0 was enriched in GABAergic markers (Fig. 5c), in contrast to cluster 1 that displayed mainly glutamatergic *Slc17a6* and *Slc17a7* transcripts (Fig. 5c). The markers *Baiap3*, *Nova1*, *Sema5a*, *Uncd5*, *Adarb2*, and *Pbx3* (typical of the *anterior* radial unit of the pallial amygdala) were present in both clusters 0 and 1, with a heterogeneous distribution (Fig. 5d). Previous results showed that *Meis1* transcripts concentrated in a distinct superficial molecular subarea of the *anterior* radial unit at cluster resolution 0.3 (Fernández et al. 2025; their Figs. 4 and 5d and S3). According to this previous analysis, this marker corresponds to neurons present selectively in the superficial stratum of the *anterior* radial pallial unit (the conventional ACo) and also in cells apparently migrated superficially into the ventral part of MeA.

Expression of the *Lhx6* gene was previously located selectively in the posterodorsal subdivision of the medial amygdala (Choi et al. 2005). We checked its amygdalar expression as well as the presence of *Lhx6* transcripts in our UMAP, noting that it appears concentrated in the GABAergic (subpallial) region of our dataset (cluster 0; Fig. 5d).

In summary, at resolution 0.15 the previous joint cluster formed by the *anterior* radial complex and the medial amygdala, can be subdivided into a GABAergic cluster (cluster 0) and a glutamatergic cluster (cluster 1). The first represents the MePD and the latter includes the *anterior* radial pallial complex and the ventral MeA (MeV), indicating a strong molecular similarity and possibly a common histogenetic origin of these two adjacent structures.

### Gene expression patterns indicate a pallial profile of the ventral medial amygdala

Transcriptomic analysis points to the existence of two functionally distinct neuronal populations in the medial amygdala. One of them is GABAergic -thus subpallial- though its cells can be separated transcriptomically from those in neighboring subpallial regions such as the central amygdala, the intercalated amygdalar complex, and caudal parts of the striatum and the pallidum (cluster 1 in Fig. 1a; MeA/ant cluster in Fig. 4c; cluster 0 in Fig. 5a). The other population is glutamatergic and is molecularly very similar to the neurons located in the *anterior* radial domain of the pallial amygdala (cluster 1 in Fig. 1a; MeA/ant cluster in Fig. 4c; cluster 1 in Fig. 5a). We checked some relevant developmental gene markers to better understand these results.

**Fig. 6:**
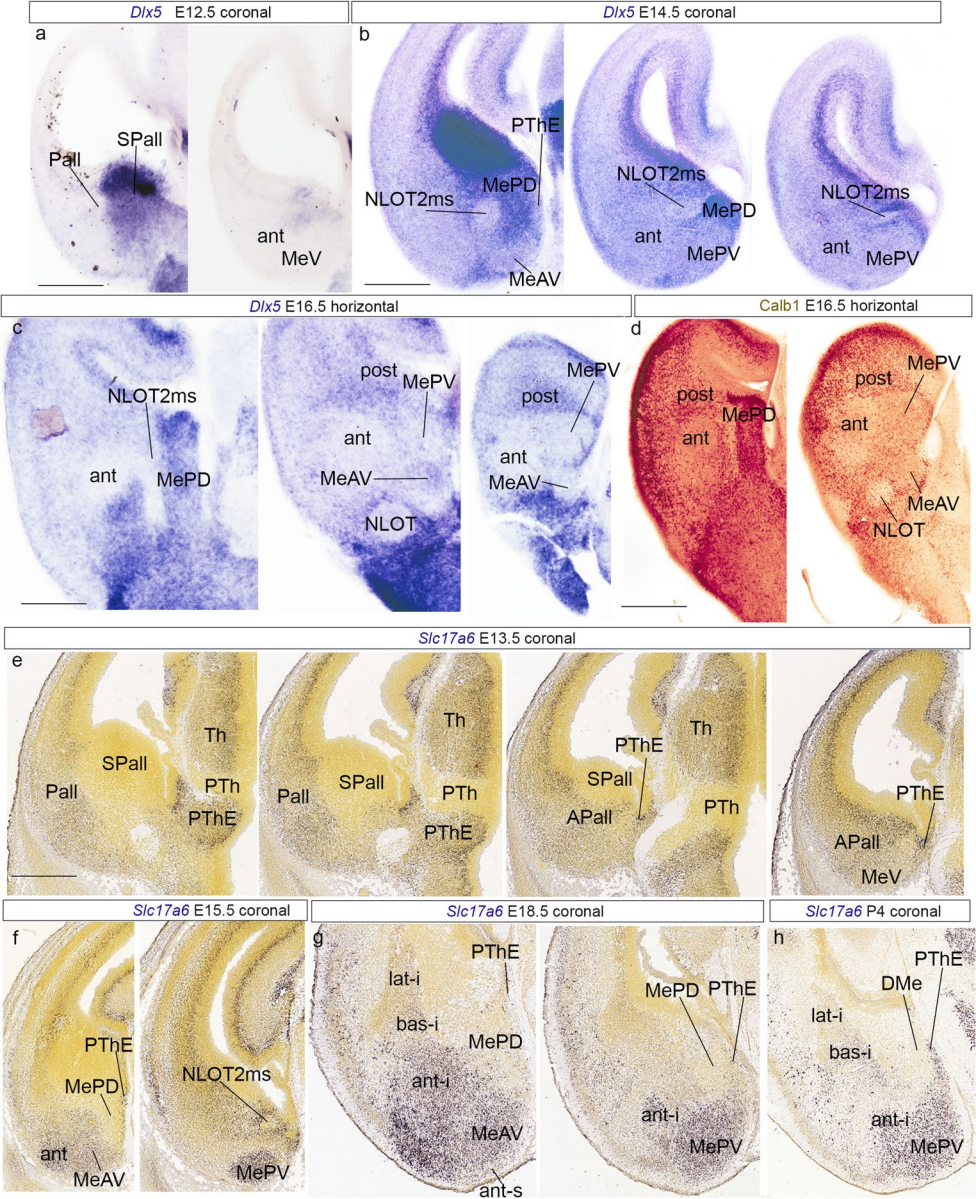
GABAergic and glutamatergic markers in the temporal telencephalic pole during development. (a) *Dlx5 in situ* hybridization at the caudal telencephalic pole, stage E12.5. Coronal plane. Scale bar 500 μm. (b) *Dlx5 in situ* hybridization at the caudal telencephalic pole, stage E14.5. Coronal plane. Scale bar 400 μm. (c) *Dlx5 in situ* hybridization at the temporal telencephalic pole, stage E16.5. Horizontal plane. Scale bar 600 μm. (d) Calbindin (Calb1) immunohistochemistry at stage E16.5. Horizontal plane. Scale bar 800 μm. (e) *Slc17a6 in situ* hybridization, at stage E13.5 downloaded from AMBA. Coronal plane. (f) *Slc17a6 in situ* hybridization, at stage E15.5 downloaded from AMBA. Coronal plane. (g) *Slc17a6 in situ* hybridization, at stage E18.5 downloaded from AMBA. Coronal plane. (h) *Slc17a6 in situ* hybridization, at stage P4 downloaded from AMBA. Coronal plane. Scale bar (E, F, G, H) 500 μm

We first analyzed the expression of *Dlx5*, a developmental subpallial marker, and *Slc17a6*, a glutamatergic marker, both known to be expressed in the area of interest. At E12.5, *Dlx5* labels generally the subpallium, being largely absent in the neighboring pallial regions, due to as yet limited tangential migration of subpallial neurons at this stage (Pall; SPall; Fig. 6a). We did not detect *Dlx5* transcripts either at the MeV primordium or at the amygdalar *anterior* radial pallial domain (ant; MeV; Fig. 6a). At E14.5, *Dlx5* transcripts were strongly present in all subpallial regions, including the MePD (Fig. 6b), but were restricted to a small number of cells identified as subpallial tangentially migrating elements in the MeV, which can be subdivided into anteroventral and posteroventral parts (MeAV; MePV; Fig. 6b). We also observed at this stage streams of *Dlx5-*positive cells apparently tangentially migrated from the subpallium into the *anterior* radial amygdalar domain. In contrast, the NLOT2 migratory stream (NLOT2ms; see Garcia-Calero et al. 2021) remains sharply negative for this marker (Fig. 6b). At E16.6, the MePD was rich in *Dlx5* transcripts and calbindin (Calb1) protein, in contrast to the MeV subdivisions (MeAV; MePV; Fig. 6c, d). The *anterior* radial domain showed MeV-like labeling (ant; Fig. 6c, d).

The glutamatergic marker *Slc17a6* labelled the pallial telencephalon at stage E13.5, including the pallial amygdala (Pall; APall; Fig. 6e). At caudal levels the positive area includes the MeV (Fig. 6e). In addition, we examined the similarly *Slc17a6* positive prethalamic eminence (PThE) at E13.5 and observed that this region contacts the caudal telencephalic pole adjacent to medial amygdalar structures (PThE; Fig. 6e). To better understand the disposition of these neural areas, we followed the expression *Slc17a6* during development of the amygdala and neighboring areas. At stage E15.5 the regions positive for this gene are the *anterior* radial domain and both MeV subdivisions (ant; MeAV; MePV; Fig. 6f). In contrast, the MePD remained negative for this marker, as occurs also with the NLOTms (Fig. 6f). The PThE abutting the amygdalar telencephalic area showed positive cells just adjacent to the negative MePD (Fig. 6f). At later developmental stages (E18.5 and P4) we observed similar expression results (Fig. 6g, h).

**Fig. 7:**
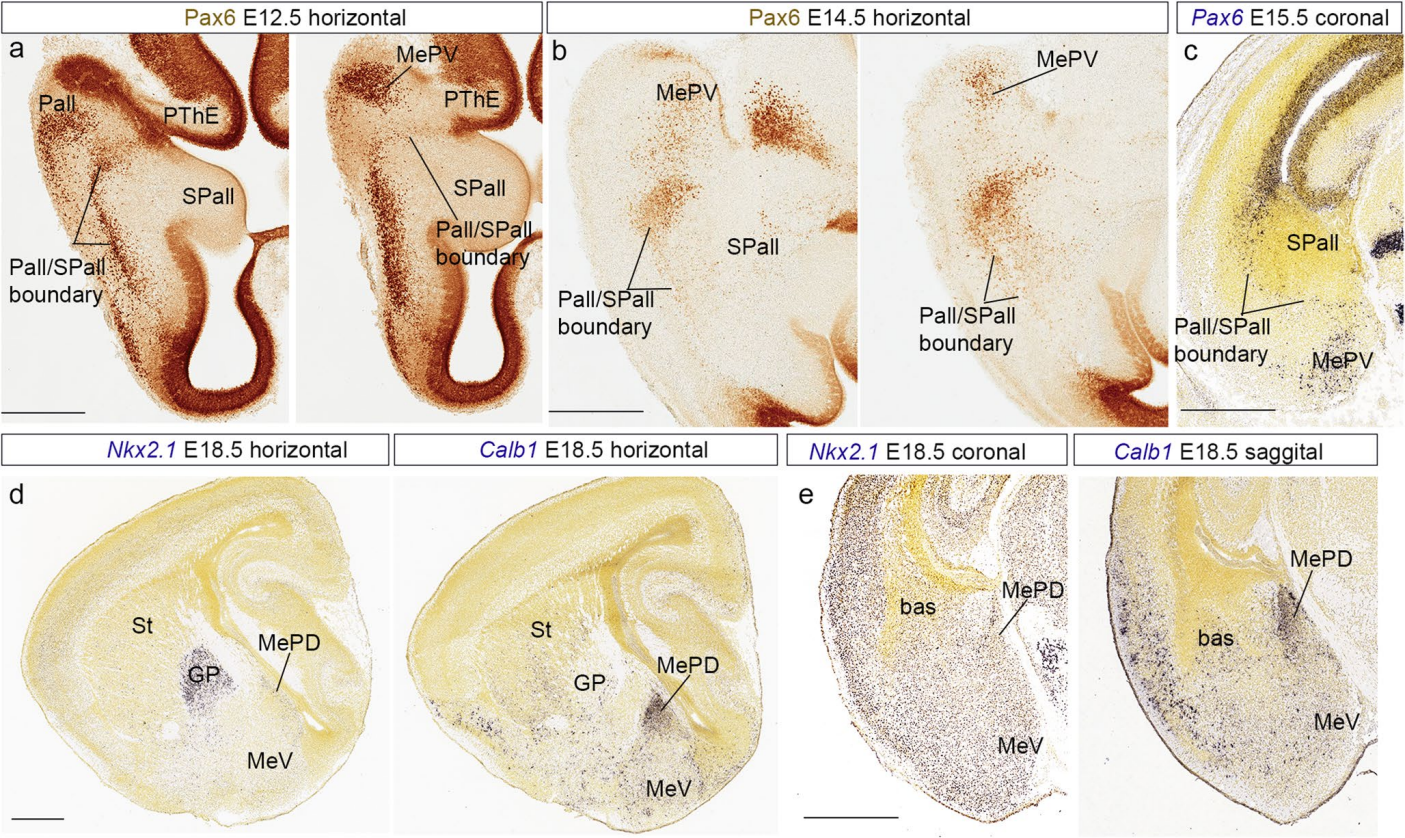
Developmental gene and protein expressions in the temporal telencephalic pole. (a) Pax6 immunohistochemistry at stage E12.5. Horizontal plane. Scale bar 400 μm. (b) Pax6 immunohistochemistry at stage E14.5. Horizontal plane. Scale bar 500 μm. (c) *Pax6 in situ* hybridization, at stage E15.5 downloaded from AMBA. Coronal plane. Scale bar 500 µ. (d) *Nkx2.1* and *Calb1 in situ* hybridization, at stage E18.5 downloaded from AMBA. Sagittal plane. Scale bar 600 μm. (e) *Nkx2.1* and *Calb1 in situ* hybridization, at stage E18.5 downloaded from AMBA. Coronal plane. Scale bar 600 μm

Next, we studied the developmental expression of the transcription factor *Pax6* at stages E12.5, E14.5 and E18.5, focusing on the amygdalar region, with immunohistochemical and in situ hybridization methods. A *Pax6*-positive radial migratory stream characterizes the pallio-subpallial boundary throughout its length (Pall/SPall boundary; Fig. 7a-c). These cells serve as a distinct linear landmark approximating the separation of the pallial and subpallial domains in the telencephalic mantle. The caudal part of this *Pax6*-positive boundary population curves into the lateral neighborhood of the MeA, leaving the MeV *outside* the pallial-subpallial boundary, i.e., apparently within the pallial region (Fig. 7a-c).

Finally, at perinatal stages (E18.5) we analyzed the preoptic markers *Nkx2*.1 and *Calb1*, and we observed the presence of positive transcripts of both genes in the MePD. These results suggest that MePD could be a derivative of the subpallial preoptic region.

These additional results are consistent with our transcriptomic data, indicating two molecularly different parts of the MeA, one - the MeV- is enriched in glutamatergic *Slc17a6-*positive neurons, shows relatively few *Dlx5*-positive elements, and seems limited by *Pax6*-positive cells from the MePD subpallial region expressing *Dlx5*. The MePD does not express any strictly pallial marker (though it shares some other markers with MeV) and displays a GABAergic profile. We thus hold that MeV is pallial, while the MePD is subpallial (moreover, it contacts laterally other subpallial subregions, such as the IcA-p, and CeA), and may be a preoptic derivative. We followed some of the markers of MeV and MePD to adult stages, and we analyzed new ones to confirm the results during development (S4). We observed that the bed nucleus of the accessory olfactory tract (BAOT) is embedded superficially within the MeAV subdivision of MeV (S4a-c). Furthermore, *Slc17a6* and *Prlr* are good markers for distinguishing respectively glutamatergic and GABAergic regions in the adult medial amygdala (S4a, d). *Irs4* gene was a marker for the preoptic region and MePD (S4e).

### Radial glia labelling DiI-experiments at the ventral MeA

**Fig. 8:**
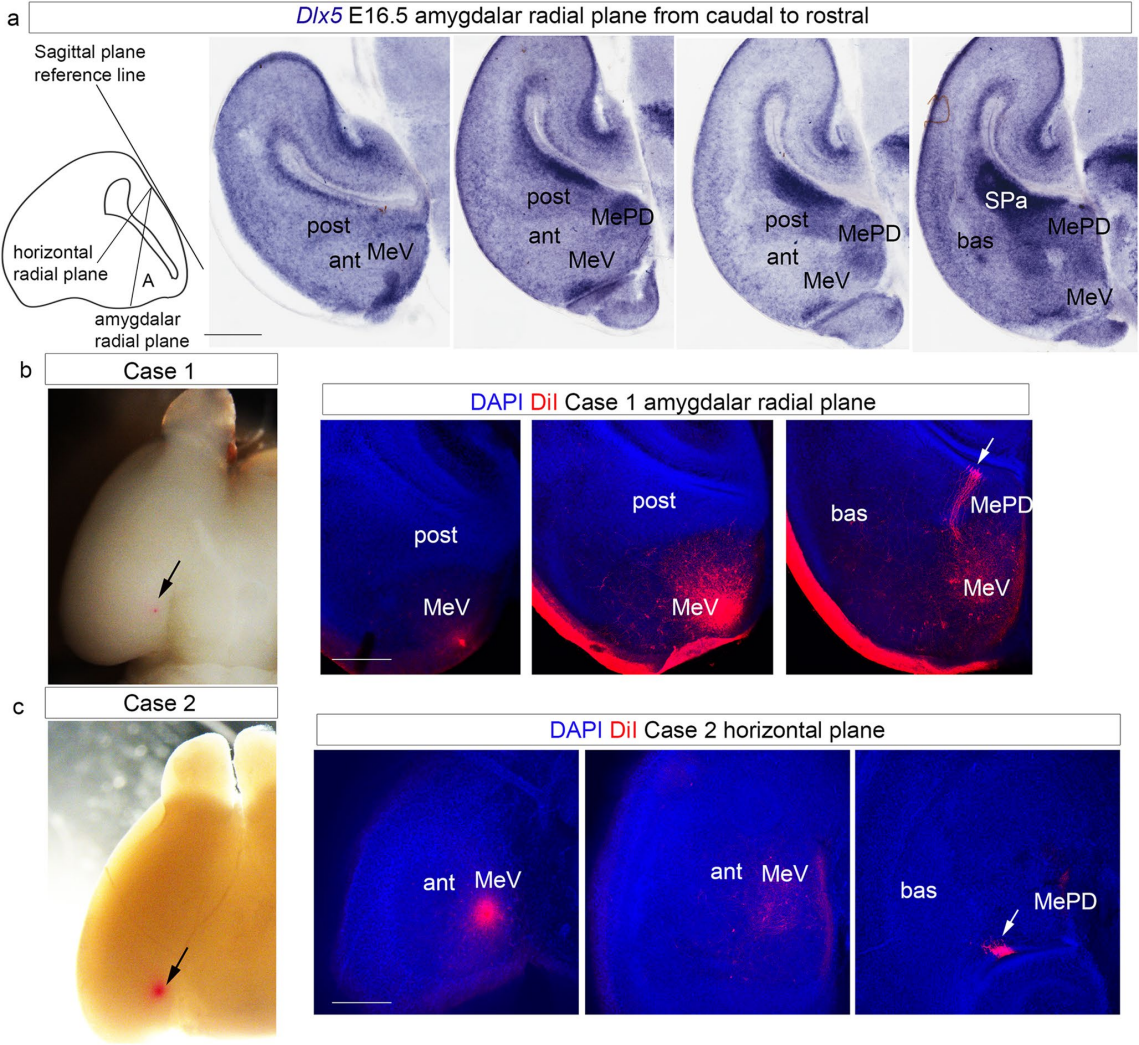
DiI experiments in the ventral medial amygdala. (a) Scheme of the dissection planes and *Dlx5* in situ hybridization at the temporal telencephalic pole, stage E16.5. Scale bar 600 μm. (b) DiI crystal inserted into the ventral medial amygdala region in an E18.5 mouse embryo. Confocal micrographs of a DiI injection at MeV, case 1, at different caudorostral levels. White arrows point to radial glial fibers in the ventricular zone. Amygdalar radial plane. Scale bar 600 μm. (c) DiI crystal inserted into the ventral medial amygdala region in an E18.5 mouse embryo. Confocal micrographs of a DiI injection at MeV, case 2, at different ventrodorsal levels. Horizontal plane. Scale bar 600 μm. White arrows point to radial glial fibers from MeV in the ventricular zone

To better understand the radial dimension of this amygdalar region (which had been excluded from our earlier analysis of radial glia in Garcia-Calero et al. 2020; under the assumption that MeA was entirely subpallial) we presently studied this point using analogous radial glia DiI-labeling experiments targeting MeV (Fig. 8). We inserted subpially DiI crystals into fixed brain specimens, aiming at several points of the surface of the MeV at E18.5/P0. After an appropriate delay, we followed the labelled radial glia processes into the ventricular area occupied by their cell bodies, as done before in Garcia-Calero et al. (2020). We also labelled again the surface of the *anterior* radial pallial unit. We analysed these results using either the *amygdalar radial section plane* (Garcia-Calero et al. 2020) or standard horizontal sections (section planes described in Fig. 8a). We compared these results with *Dlx5* expression at E16.5, to better characterize the route of radial glia bundles, following them from caudal to rostral in amygdalar radial plane cases or ventral to dorsal in horizontal sections (Fig. 8).

Placement of a DiI microcrystal at the MeV pial surface at E18.5/P0 (*n* = 4; black lines in Fig. 8b, c) labelled selectively a radial glia bundle that crossed radially through either the MePV (caudally) or the MeAV (rostrally) to end in a ventricular zone lying *lateral* to the subpallial (*Dlx5* positive) MePD area, rostrally to the periventricular area that belongs to the *posterior* pallial radial unit (post, Fig. 8; white lines in Fig. 8b and c point to radial glia fibres in the ventricular region). All radial glia bundles marked from the MeV area eschew the MePD subdivision, a feature that is best observed in amygdalar radial plane sections (Fig. 8b). The MeV ventricular region was interpreted as subpallial in Garcia-Calero et al. (2020), but present results incline us to postulate its pallial nature.

## Discussion

The main results of this study were: (a) there is a close molecular similarity of the ventral medial amygdala (MeV) with the neighbouring *anterior* radial domain of the pallial amygdala, as determined by transcriptomic analysis and a mainly glutamatergic neuronal population; (b) the MeV is an area poor in subpallial markers that possesses an independent radial glial structure associated to the pallial amygdala. These data suggest dividing the classic medial amygdala into a posterodorsal *subpallial* component (MePD) and a ventral *pallial* component (MeV) encompassing the conventional MePV, MeAV and MeAD areas.

The Results indicated two major amygdalar clusters and three minor ones (Fig. 4c): one of the major clusters (‘MeA/ant’) represents the whole medial amygdala plus the glutamatergic *anterior* radial amygdalar pallial unit, while the other (‘lat/bas/post’) contains the rest of the pallial amygdala (*lateral*, *basal* and *posterior* amygdalar radial units). The minor clusters represent the central amygdala (‘CeA’), the intercalated amygdalar nuclei (‘IcA’) and the Sim1-positive population (‘Sim1’).

The fact that the profile of the ‘MeA/ant’ cluster lies closer molecularly to the cluster with (subpallial) central amygdalar neurons than the ‘lat/bas/post’ cluster was interpreted as due to the presence inside ‘MeA/ant’ of the largely GABAergic cells of the MePD; otherwise, the MePD and MeV share various gene markers. The UMAPs (Fig. 1c) revealed a polarization of the ‘MeA/ant’ cluster into largely GABAergic-rich and glutamatergic-rich subfields.

Subsetting of the ‘MeA/ant’ cluster obtained distinct clusters; one enriched in *Slc17a7* and *Slc17a6* (glutamatergic markers) and another enriched in *Gad1*, *Gad2* and *Lhx6* (GABAergic markers). Glutamatergic markers were absent from the MePD, where *Gad2* is markedly present. Interestingly, only *Slc17a6* labelled importantly the MeV as well as the *anterior* pallial radial amygdalar unit, whereas *Slc17a7* characterized instead the separate ‘lat/bas/post’ amygdalar cluster. The gene *Baiap3* and various other markers nevertheless appears expressed in both MeA subdivisions, as well as in the *anterior* radial pallial unit. This reveals the existence of some shared molecular determinants across the two MeA parts (perhaps due to a shared cell population migrated from the adjacent prethalamic eminence, as suggested by expression of the marker *Neurod1*; Allen Mouse Developing Brain Atlas).

### The IcA component

We also characterized another subpallial amygdalar cluster as containing selectively populations of the intercalated amygdalar islets (IcA: Fig. 4c). This nuclear complex is poorly described in the literature. After our genoarchitectural analysis with various selective markers we describe it as a network of islands of different sizes distributed all around the frontal surface of the rostrally protruding lateral and basal amygdalar nuclei, along the curved striato-amygdalar boundary and at the back of the central nucleus (in sagittal sections). In coronal sections the IcA appears ventral and ventromedial to CeA, ending periventricularly (IcA-p) next to the MePD nucleus (IcA-p; Fig. 2b, S2b). This deep area contains fibres of the stria terminalis and is commonly interpreted as the STIA (amygdalar nucleus of the stria terminalis; Paxinos and Franklin 2019). The largest IcA island appears typically ventral to the rostral pole of the classic BLA nucleus (our bas-i in the present work; sometimes it is identified as the ‘Main IcA’ nucleus). In García-Calero et al. (2020) we interpreted that the IcA population results stretched developmentally by the massive rostral protrusion of the lat/bas radial units (e.g., see in coronal AMBA data at E18.5 the *Foxp2* expression at the IcA primordium; not shown here).

The subpallial amygdalar structures (CeA, IcA, and MePD) are molecularly and topographically close to each other, though each of them also has some differential markers.

### A bipartite MeA

The conventional notion of the mammalian MeA conceives it as a unitary subpallial medial amygdalar region (Krettek and Price 1978; De Olmos et al. 1985; Olmos et al. 2004; Alheid et al. 1995; Martinez-García et al. 2012; Olucha-Bordonau et al. 2015; Medina et al. 2017). Cytoarchitectonic, chemoarchitectonic and neurotransmitter studies have fundamented the involvement of the MeA in the vomeronasal sensory system and uncovered specific hypothalamic projections (Krettek and Price 1987; Swanson and Petrovich 1998; Gutierrez-Castellanos et al. 2014).

We have shown that the MeA is actually subdivided into a caudally and largely periventricular subpallial *posterodorsal part* (MePD), found laterally to the stria terminalis tract next to the insertion of the chorioidal tela in the medial amygdala and limiting caudally with the prehippocampal medial extension of the *posterior* amygdalar pallial complex (amygdala-hippocampal, AHi, and posteromedial cortical, PMCo, nuclei), and a relatively rostral and more superficial pallial *ventral part* (MeV). We suggest in the present work that due to the expression of *Nkx2.1* and *Calb1* (also see *Irs4* expression) in MePD, this region possibly is a derivative of the subpallial preoptic region.

Significantly, our DiI experiments indicate that the radial glia fibres of the MeV eschew the MePD ventricular area and consistently reach a previously unrecognized *Dlx5*-negative ventricular area intercalated between the MePD and the posterior pallial radial complex. If the MePD represented the periventricular stratum of the MeV, as was previously assumed, the radial glia pattern should have united them into a single radial domain.

The MeV, and particularly its posterior part, has been the focus of different studies emphasizing its heterogenous cell population, due to a remarkable high content of non-GABAergic cells (around 70% of the local population; Keshavarzi et al. 2014). Our results mapping *Dlx5* expression and Calb1 marker over several developmental stages also indicate a low number of GABAergic cells in the MeV. The neuronal heterogeneity of MeV can be partly explained in terms of tangential migratory translocation of cell populations from other regions, as was advanced in the Introduction (Hirata et al. 2009; Carney et al. 2010; Lischinsky et al. 2017; Puelles et al. 2016; Ruiz-Reig et al. 2018). An alternative explanation might consider a primary majoritarily subpallial cell population that is secondarily massively invaded by tangentially migrated glutamatergic cells. There is indeed some evidence of immigrating *Lhx9*-positive pallial cells from the *anterior* pallial radial unit (García-López et al. 2008; Garcia-Calero and Puelles 2021), but the apparent magnitude of this migration is not sufficient to justify 70% of pallial elements in the MeV. Our radial glia experiments indicating at E18.5/P0 a location of the MeV radial glia cell bodies in a *Dlx5*-negative pallial ventricular zone seems consistent with the first interpretation, which ascribes to MeV a fundamental pallial nature associated to minor invasion of subpallial cells (as well as of some *Lhx9*-positive anterior amygdalar pallial cells). There are also separate contingents of tangentially migrated *Shh*-, *Otp*-, and *Sim1*-positive cells coming from the preoptic area and alar hypothalamus (Carney et al. 2010; Garcia-Moreno et al. 2010; Garcia-Calero et al. 2021) via the hypothalamo-amygdalar corridor described by us under the sulcus terminalis (Garcia-Calero et al. 2021). In addition, in the present work we described the BAOT nucleus, which appears located superficially within MeAV (S4). We thus deduce the BAOT also may be a pallial component of MeV.

Considering all relevant results (Garcia-Calero et al. 2020; Fernandez et al. 2025, present results), the MeV is an amygdalar pallial component that is independent from the subpallial MePD, and there are two interpretive possibilities: (a) it may represent an *additional* amygdalar pallial radial unit in the neighbourhood of the *posterior* and *anterior* units; however, the considerable molecular similarity of the MeV and the population of the *anterior* radial unit argues against this option, or (b) it may form a medial part of the *anterior* pallial amygdalar radial unit; this option seems supported by their considerable transcriptomic molecular similarity detected in the present study.

As regards the MePD, it is transcriptomically neatly distinguishable from the MeV and shows similarity (but not strong identity) with various other subpallial amygdalar formations (i.e., the neighbouring IcA and CeA domains; Figs. 6 and 7). Its distinctness can be due to a possible preoptic or diagonal origin, suggested by our *Nkx2.1* and calbindin data (also *Irs4* in S4e) and the course of the stria terminalis tract.

## Materials and methods

### Animals

Experimental protocols were approved by the Committee for Animal Experimental Ethics (University of Murcia) and CARM (Autonomous Community of the Region of Murcia; No. A13230704). Male and female adult mice (C57BL/6 strain) were sacrificed at the Animal Housekeeping Facility, CEIB, University of Murcia: animals were first anesthetized with 4–5% isoflurane and subsequently decapitated. Mouse embryos were obtained from pregnant females after their sacrifice. Brains from embryos and adult mice were extracted and subsequently processed.

### SnRNA-seq data processing of subpallial and pallial amygdala data

The initial dataset of the present work derived from the single-nuclei RNA sequencing data analysis published in Fernandez et al. (2025). The material in Fernandez et al. (2025) was obtained by dissecting the amygdala region and neighboring telencephalic regions from four adult mice, two males and two females.

Quality filters applied to this dataset were: number of genes per cell minor than 2.500, number of UMIs per cell major than 500 and minor than 5.000, and percentage of mitochondrial RNA minor than 0.5%. We subsetted the GABAergic clusters and glutamatergic amygdala clusters, annotated as GABA and APall in the initial Seurat object of 31.848 nuclei, Fig. 2e in Fernandez et al. (2025), and obtained a new dataset of 6.979 high quality nuclei containing the amygdalar GABA and APall clusters. We applied the Seurat workflow to this dataset: the “FindVariableFeatures” (selection.method = “vst”) function was used to detect highly variable genes among the nuclei; we selected 3000 highly variable features. The object was scaled (“ScaleData” function; regressed variables applied were “nCount_RNA”, “nFeature_RNA” and “percent.mt”. A Principal Components (PCA) study was done on our scaled data. An Elbow (first 50 PCs) was analyzed, and after careful testing, we used the first 30 PCs for the next analysis. We applied the “FindNeighbors” and “FindClusters” functions (Louvain clustering) to our dataset. For cell clustering we tested different resolutions: 0.05, 0.1, 0.15, and 0.2. These results were reduced using Uniform Manifold Approximation and Projection for Dimension Reduction (UMAP) plot (Fig. 1a). The number of clusters obtained was 10.

We used violin plots and scatter plots to check out the metrics by samples distribution (Features/Genes per nucleus; Counts/UMIs per nucleus; percentage of mitochondrial RNA per nucleus), correlations between metrics (genes vs. UMIs per nucleus). The results indicate adequate characteristics (number of genes and UMIs, low mitochondrial percentage). The 10 clusters were represented in the four samples (S1b).

### SnRNA-seq data processing of the anterior radial complex and medial amygdala dataset

We subsetted cluster 1 (Fig. 1a; cluster annotated as MeA/ant in Fig. 4c) from the initial pallial amygdala and GABAergic-Seurat object. The Seurat workflow was repeated with these parameters: 30 PCs for dimensionality and different cluster resolutions were checked (S3). The clustering results were visualized with UMAP plots (S3), and the optimal resolution was 0.15. Nuclei from four samples were present (Fig. 5a and b). For cluster annotation we used GABAergic and glutamatergic markers, markers of the *anterior* radial unit published recently (Fernandez et al. 2025), and *Lhx6* as an additional marker of the medial amygdala (Fig. 5c-f).

### RNAscope in situ hybridization

In situ hybridization was performed using RNAscope Fluorescent Multiplex Kit (RNAscope^®^ Intro Pack for Multiplex Fluorescent Reagent Kit v2- Mm with TSA Vivid Dyes- RNAscope^®^, Advanced Cell Diagnostics, Cat. 323280) following the manufacturer’s instructions for fresh tissue. The protocol used was published in Fernandez et al. (2025). RNAscope probes used in the present work were: Mm-*Baiap3-*C3 (Cat. 1133511-C3), Mm-*Gad2*-C4 (Cat. 439371-C4), Mm-*Slc17a6*-C2 (Cat. 319171-C2), RNAscope Probe – Mm-*Slc17a7* (Cat. 416631).

### In situ hybridization

The hybridization protocol used was according to Shimamura et al. (1994). For the riboprobe preparation we used restriction enzymes and polymerases in the presence of digoxigenin-11-UTP. The mouse cDNA probe used for in situ hybridization analysis was *Dlx5* (J.R. Rubenstein lab).

### Immunohistochemistry

For immunostaining, we followed the protocol published in Garcia-Calero and Scharff (2013). The primary antibodies used in this study were: rabbit anti-calbindin (1:1000; CB-38, Swant, Bellinzona, Switzerland), and rabbit anti-Pax6 (1:500; Covance Catalog number PRB-278P).

### DiI experiments

Embryonic brains at stage E18.5, and postnatal days P0 were dissected out and fixed 24 hours in 4% paraformaldehyde in pH 7.4 PBS at 4°C. Small DiI crystals (1,1’-dioctadecyl 3,3,3’,3’-tetramethylindocarbocyanine perchlorate; Molecular Probes) were inserted with a sharpened tungsten needle into the pial surface of the medial amygdala and DiI was allowed to diffuse at 37 °C during 15 days. After this time, the brains were cut on a vibratome into 120 μm sections and counterstained with DAPI (1/1000; Sigma).

### Imaging

Digital microphotographs were acquired using a widefield microscope (Leica Thunder-imager) with a 20x objective (processed using a proprietary background subtraction methodology, Leica ICC, to improve the results), and digital cameras DC500 or DC350 (Leica, Wetzlar, Germany) and a confocal microscope (TCS SP8 AOBS; Leica Microsystems GmbH, Mannheim, Germany). The images (z-stacks) were acquired with the LCS software. The digital images were processed with ImageJ (NIH, https://rsb.info.nih.gov/ij), Adobe Photoshop and Adobe Illustrator software (Adobe Systems MountainView, CA, USA).

### AGEA tool and in situ hybridization figures from Allen adult mouse brain atlas

We used the AGEA data-mining function in the AMBA (https://mouse.brain-map.org/agea ) and we downloaded in situ hybridization figures for the selected genes from the Allen Mouse Brain Atlas (RRID: SCR_017001; URLs: http://portal.brainmap.org ).

## Supplementary Information

Below is the link to the electronic supplementary material.

Supplementary Material 1

## Data Availability

No datasets were generated or analysed during the current study.

## Abbreviations

ACx: Allocortex
ant-i: Anterior radial domain, intermediate stratum
ant-s: Anterior radial domain, superficial stratum
bas-i: Basal radial domain, intermediate stratum
CeA: Central amygdala
EPD: Endopiriform nucleus
ERh: Entorhinal cortex
GP: Globus pallidus
Hi: Hippocampus
IcA: Intercalated nucleus
IcA-i: Intercalated nucleus, intermediate stratum
IcA-p: Intercalated nucleus, periventricular stratum'
lat/bas/post: Lateral/basal and posterior amygdalar radial domains
lat-i: Lateral radial domain, intermediate stratum
MeA: Medial amygdala
MeAV: Anteroventral medial amygdala
MCx: Mesocortex
MePD: Posterodorsal medial amygdala
MePV: Posteroventral medial amygdala
MeV: Ventral medial amygdala
NLOT2: Nucleus of the lateral olfactory tract, layer 2
NLOT2ms: Nucleus of the lateral olfactory tract, layer 2, migratory stream
opt: Optic tract
Pall: Pallium
Pir: Piriform cortex
PO: Preoptic region
post: Posterior radial domain
PthE: Prethalamic eminence
SPall: Subpallium
STh: Subthalamic nucleus
St: Striatum
Th: Thalamus
