# Supplementary material for: Transcriptomics reveals pallial and subpallial subdivisions of the mouse medial amygdala
Source: Brain Struct Funct. 2026 Feb 2;231(2):20. doi: 10.1007/s00429-026-03076-x (PMC12864216; doi:10.1007/s00429-026-03076-x)
Supplement: Supplementary file 1 — Supplementary Material 1 [file 429_2026_3076_MOESM1_ESM.docx]

**Supplementary Information**

**Transcriptomics reveals pallial and subpallial subdivisions of the mouse medial amygdala**

Gloria Fernández^1^, Luis Puelles^1^, Eduardo Pons-Fuster^1^, Ramón Pla^1^, Elena Garcia-Calero^1*^

^1^ Departamento de Anatomía Humana y Psicobiología, Facultad de Medicina, Universidad de Murcia e Instituto Murciano de Investigación Biosanitaria IMIB-Pascual Parrilla, Murcia, Spain.

*Corresponding author:

e-mail:ecalero@um.es

https://orcid.org/0000-0002-7184-9584

**S1**

**
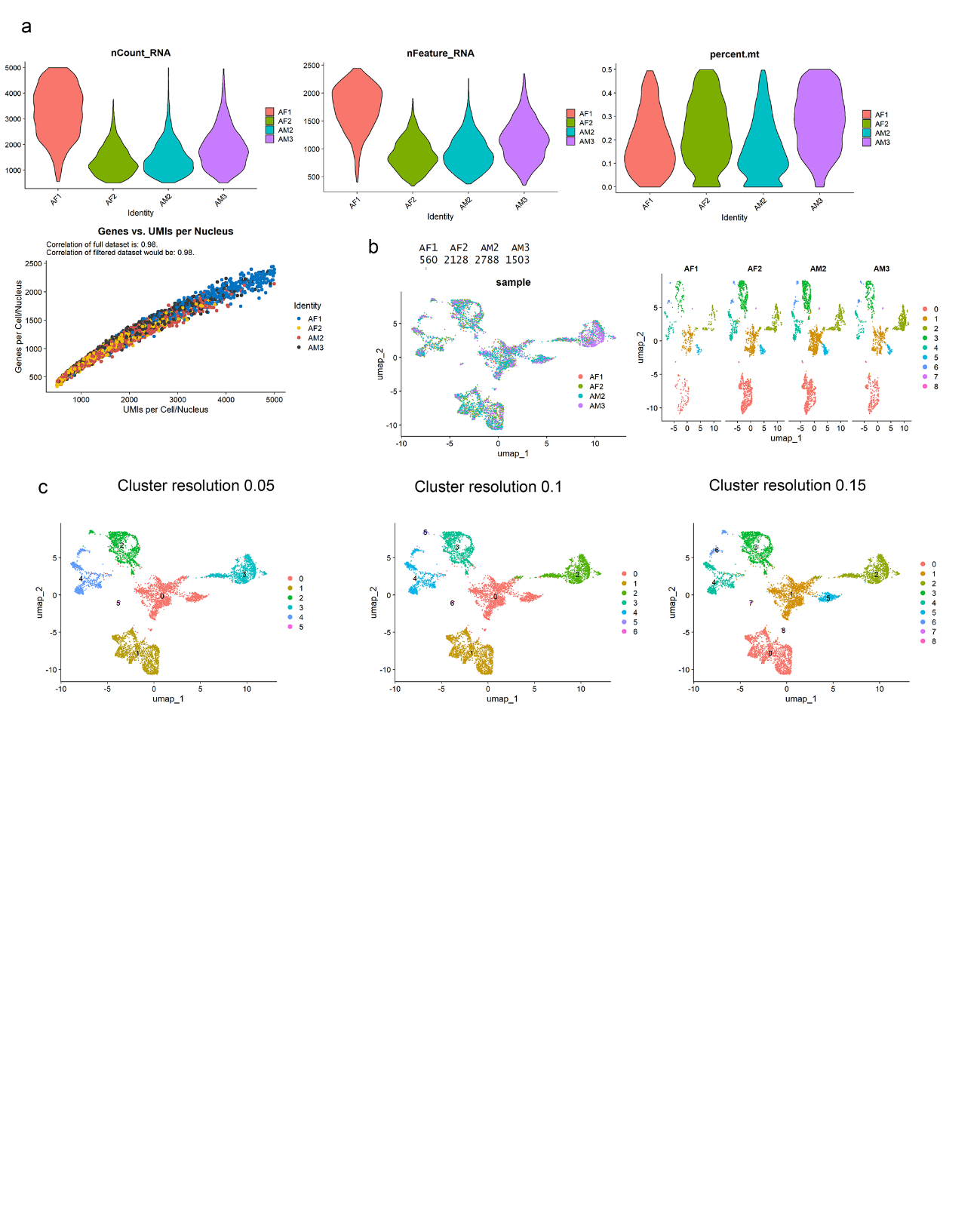
**

**Quality control and cluster resolution of pallial amygdala and GABAergic-Seurat object**

(a) Violin plots showing counts (UMIs) per nucleus, features (genes) per nucleus, mitochondrial percentage per nucleus of the pallial amygdala and GABAergic-Seurat object (6.979 nuclei), split by sample. Scatter plot showing correlations between genes split by sample. (b) UMAP plots of the pallial amygdala and GABAergic-Seurat object group by sample and split by sample. (c) UMAP plots of pallial amygdala and GABAergic-Seurat object at cluster resolutions 0.05, 0.1 and 0.15.

**S2**

**
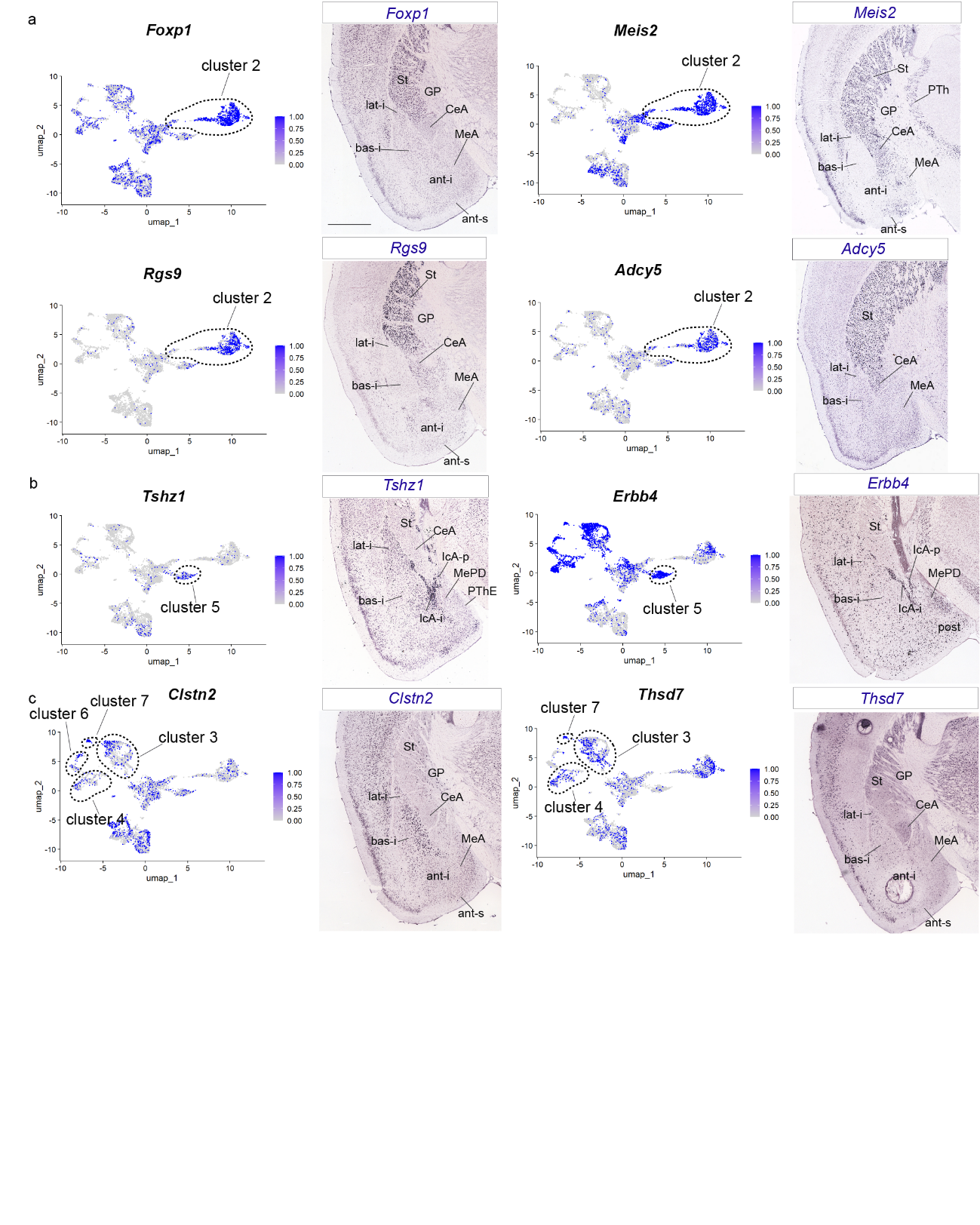
**

**Gene markers for striatum, intercalated amygdala nuclei and pallidal region**

(a) UMAP plots for *FoxP1,* *Meis2, Rgs9* and *Adcy5* of the pallial amygdala and GABAergic-Seurat object, and *in situ* hybridization for these genes. Cluster 2 was highlighted with an outline in the UMAP plots. (b) UMAP plots for *Tshz1 and Erbb4* of the pallial amygdala and GABAergic-Seurat object, and *in situ* hybridization for these genes. Cluster 5 was highlighted with an outline in the UMAP plots. (c) UMAP plots for *Clstn2* and *Thsd7* of the pallial amygdala and GABAergic-Seurat object, and *in situ* hybridization for these genes. Clusters 3, 4, 6 and 7 were highlighted with an outline in the UMAP plots. The *in situ* hybridization figures of Fig. 3 were downloaded from AMBA. Coronal plane. Scale bar 930 µm.

ant-i; *anterior* radial complex, intermediate stratum; ant-s: *anterior* radial complex, superficial stratum; bas-i: *basal* radial complex, intermediate stratum; CeA: central amygdala; GP: globus pallidus; IcA-i: intercalated nucleus, intermediate stratum; IcA-p: intercalated nucleus, periventricular stratum; lat-i: *lateral* radial complex, intermediate stratum; MeA: medial amygdala; MePD: posterodorsal medial amygdala; PthE: prethalamic eminence; St: striatum.

**S3**

**
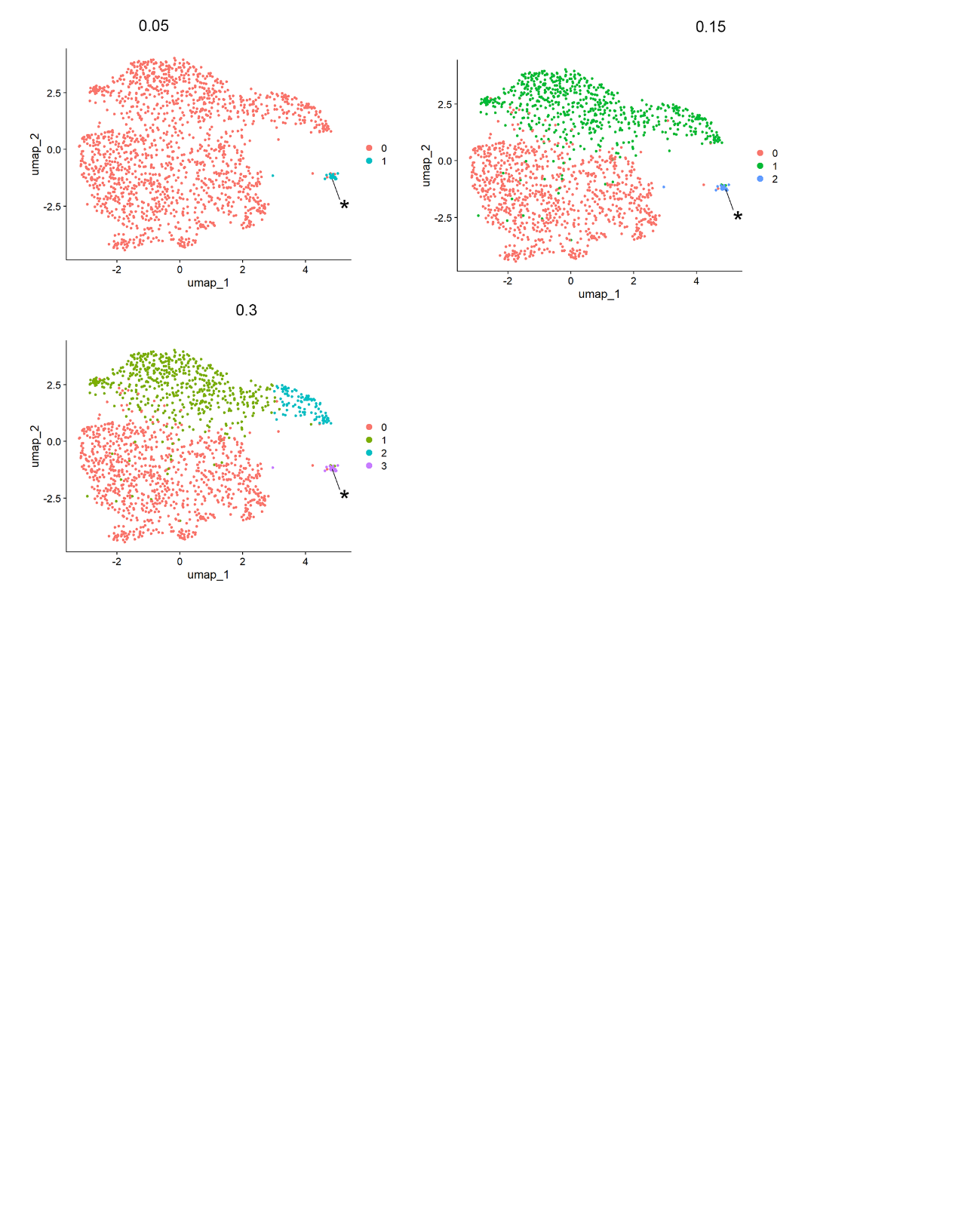
**

**Cluster resolution for *anterior* radial unit and MeA-Seurat object**

UMAP plots of the *anterior* radial unit and MeA-Seurat object at cluster resolutions 0.05, 0.15, 0.3.

**S4**

**
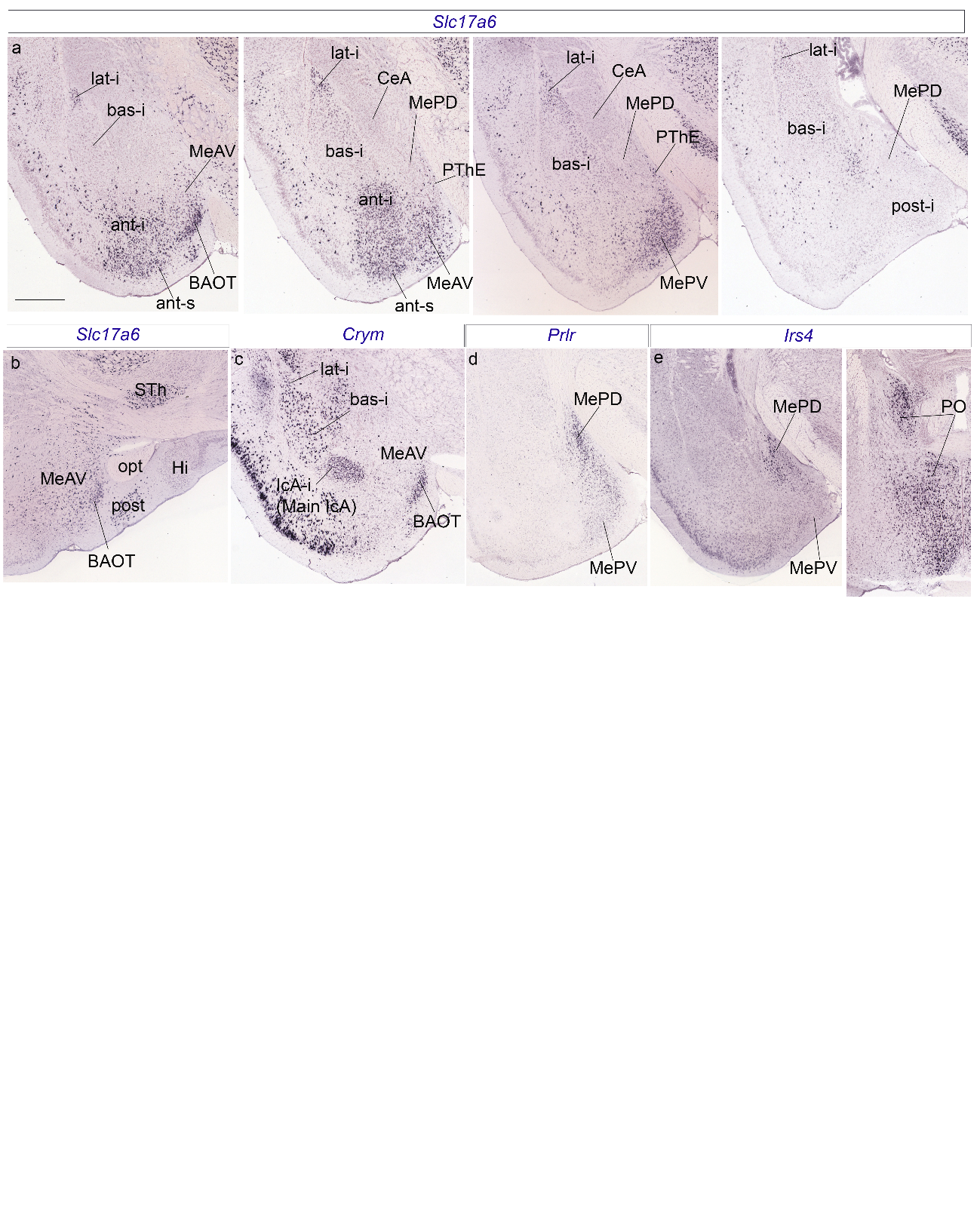
**

**Gene markers for medial amygdala and neighboring regions at adult stages**

(a) *Slc17a6 in situ* hybridization, at stage P56, downloaded from AMBA. Coronal plane. (b) *Slc17a6 in situ* hybridization, at stage P56, downloaded from AMBA. Sagittal plane. (c) *Crym in situ* hybridization, at stage P56, downloaded from AMBA. Coronal plane. (d) *Prlr in situ* hybridization, at stage P56, downloaded from AMBA. Coronal plane. (e) *Irs4 in situ* hybridization, at stage P56, downloaded from AMBA. Coronal plane. Scale bar 600 µm.

ant-i; *anterior* radial complex, intermediate stratum; ant-s: *anterior* radial complex, superficial stratum; BAOT: accessory olfactory tract bed nucleus; bas-i: *basal* radial complex, intermediate stratum; CeA: central amygdala; IcA-i: intercalated nucleus, intermediate stratum; lat-i: *lateral* radial complex, intermediate stratum; MeA: medial amygdala; MeAV: anteroventral medial amygdala; MePD: posterodorsal medial amygdala; MePV: posteroventral medial amygdala; PthE: prethalamic eminence.
